# Paraneoplastic Mononeuritis Multiplex as a Presenting Feature of Adenocarcinoma of the Lung

**DOI:** 10.1155/2013/457346

**Published:** 2013-12-23

**Authors:** Esra Ekiz, Abdullah Ozkok, Nazan Kader Ertugrul

**Affiliations:** ^1^Haskoy State Hospital, Department of Internal Medicine, 49700 Mus, Turkey; ^2^Department of Nephrology, Istanbul Medeniyet University, Goztepe Training and Research Hospital, 34710 Istanbul, Turkey; ^3^Eruh State Hospital, Department of Family Medicine, 56800 Siirt, Turkey

## Abstract

Paraneoplastic neurologic syndromes are observed in less than 0.1% of cancer patients. Neurologic syndromes in lung cancer include Lambert-Eaton myasthenic syndrome, polyneuropathy, cerebellar degeneration, and rarely mononeuritis multiplex. In this case, a patient presenting with bilateral asymmetrical sensorimotor polyneuropathy who was diagnosed with adenocarcinoma of the lung is reported.

## 1. Introduction

Mononeuritis multiplex is a painful, asymmetrical, asynchronous sensory, and motor peripheral neuropathy involving isolated damage to at least 2 separate nerve areas. Multiple nerves in random areas of the body can be affected. As the condition worsens, it becomes less multifocal and more symmetrical. Mononeuropathy multiplex syndromes can be distributed bilaterally, distally, and proximally throughout the body [1, 2].

The condition is associated with (but not limited to) systemic disorders such as diabetes, vasculitis [3], amyloidosis, direct tumor involvement, lupus, paraneoplastic syndromes, and some viral infections including AIDS [4].

## 2. Case Report

A 65-year-old male attended to the hospital with a one-month-long history of pain in both legs. The pain was burning in nature, 7-8/10 in intensity (on a scale of 1 to 10, with 10 being the worst pain), lasting for more than half of the day, including night time and resting. There was also tingling sensation on both soles. The patient had difficulty walking, since the symptoms started.

He complained of some loss of appetite, without any weight loss. No headache or visual disturbances were seen. He neither had dyspnea nor night sweats. Inspite of having severe pain in both legs, he hardly ever had loss of muscle strength.

The patient was a smoker, with a 30 pack/year history of smoking. He had had an extracorporeal shock wave lithotripsy (ESWL) 3 years ago for a renal calculus in his left kidney. His family history was unremarkable.

Physical exam was normal except for a fever of 38.3 degrees celsius and a mild decrease in muscle strength in lower extremity (4/5 bilaterally). The patient had a white blood cell count of 19000, with neutrophil predominance (87% polymorphonuclear cells) and a normochromic normocytic anemia (Hct: 29.8%, MCV: 88, MCHC: 32). He had a 20-fold increase in C-reactive protein (CRP) and a sedimentation rate of 139 mm/hr. Routine biochemical tests showed a mild decrease in albumin levels (Alb: 2.4 gr/dL), which could have been interpreted as a negative acute phase reaction. His creatinine was also mildly increased up to 1.8 mg/dL.

Urinalysis revealed numerous erythrocytes and few leukocytes, with 1+ proteinuria. The patient was started on parenteral ceftriaxone for pyelonephritis, possibly caused by recurrent nephrolithiasis, after drawing blood and urine cultures. After 48 hours on ceftriaxone, there had been no decrease in fever, and antibiotherapy was switched to piperacillin-tazobactam.

For the pain management of the patient, tramadol was preferred, due to his decreased GFR. In five-day time, neuropathic pain was also involved in his upper extremity and this ascending type of neuropathy raised the suspicion of either Guillan Barré Syndrome or syphilitic spinal cord involvement. His VDRL-RPR and TPHA tests were negative, along with a negative serology for Parvo B19, CMV, EBV, HSV, HIV, and hepatitis viruses. Lumbar puncture did not reveal albuminocytologic dissociation either. EMG showed mixed type multifocal polyneuropathy, asymmetrically involving both upper and lower extremity motor and sensory fibers.

There was no electrolyte imbalance, which might possibly explain the sensory and motor neuropathy. Since the patient was a smoker, he had a routine chest X-ray, which revealed a solitary mass in the apical region of his left lung. He was not asthmatic and did not have involvement of the nose or nasal septum, and also was negative for ANA and ANCA; thus, Wegener's and Churg Strauss were excluded.

With the patient being hematuric and proteinuric and having fever with an elevated ESR, his 24 hr urine study showed a protein excretion of 1.35 gr/day with a calculated GFR of 40 mL/min. He had both serum protein and urine immunofixation electrophoreses, the results of which were within normal ranges.

**Figure 1:**
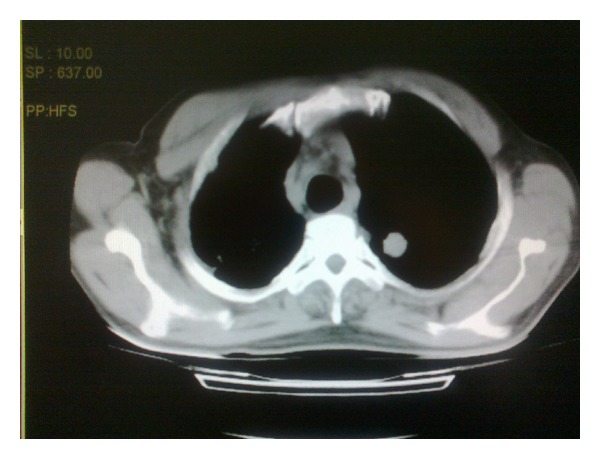
Nodular lesion located at the apex of the left lung.

On followup, he started to complain of low back pain. Brucellosis was ruled out with both qualitative and quantitative tests. After all, he had a whole spinal magnetic resonance imaging (MRI) in order to exclude a possible plasmcytoma and/or a solid tumor metastasis to the vertebrae. No mass lesion was seen. The patient had thoracic and abdominal computerized tomography (CT), in order to see if there were any lymphadenopathies. Abdominal CT was unremarkable, whereas CT of thorax showed a 3 cm nodule with irregular borders and spicules, localized in the left apex (Figure 1). This was highly suspicious of a malignant lesion. After that, the lesion was biopsied under the guidance of CT, which was reported as adenocarcinoma. The patient had a positron emission tomography (PET) scan and PET showed a left apicoposterior malignant lesion with multiple metastatic lymphadenopathies in the mediastinum. There was pathologic FDG signalling in the spleen and iliac bone marrow. Thus, the patient had a bone marrow biopsy, which revealed diffuse carcinoma metastasis. He was then transferred to oncology for chemotherapy.

This patient was diagnosed with primary adenocarcinoma of the lung with paraneoplastic mononeuritis multiplex as the presenting feature. Adenocarcinoma of the lung rarely metastasizes to the bone marrow and the primary symptom being paraneoplastic peripheral neuropathy makes the case remarkable.

## 3. Discussion

Paraneoplastic neuropathies (PN) are rare, affecting approximately 4-5% of patients with cancer. Their diagnosis is difficult, because the clinical picture is nonspecific and the neuropathy precedes the discovery of the cancer in a majority of patients [5].

In a study ran by Živković et al., mononeuritis multiplex was found to be the most common form of paraneoplastic neuropathy, with a ratio of 27.5% versus 4% in controls [6]. This form of neuropathy is usually associated with hematologic malignancies. Small cell lung cancer is known to be the most common solid tumor which is associated with paraneoplastic neuropathies [7]. However, mononeuritis can rarely be seen with nonsmall cell lung cancer (NSCLC), too. Martin et al. reported a case of NSCLC who developed mononeuritis multiplex shortly after the diagnosis of the malignant tumor [8]. Our case, being also a nonsmall cell lung cancer, was different from previously reported cases as mononeuritis was the presenting symptom before the patient was diagnosed with adenocarcinoma.

In a review of 52 cases with several solid tumors, including small cell lung cancer, nasopharyngeal cancer, and hepatocellular carcinoma, the duration of the peripheral neuropathy varied from 2 to 11 months before the malignant tumors were confirmed [9]. Our patient also had 2 months of neuropathic pain before the definitive diagnosis was made.

## 4. Conclusion

Although being rare, mononeuritis multiplex can be a presenting symptom of nonsmall cell lung cancer. Unexplained neuropathies should thus raise the suspicion of an underlying malignancy. Adenocarcinoma of the lung should also be kept in mind in such cases, as it can be manifested with peripheral neuropathy as the cardinal symptom.
